# Adrenal Incidentaloma Controversial Size Recommendations

**DOI:** 10.5152/tud.2023.22245

**Published:** 2023-03-01

**Authors:** Brandon S. Jackson

**Affiliations:** Department of Surgery, Kalafong Provincial Tertiary Hospital and Faculty of Health Sciences, University of Pretoria, South Africa

**Keywords:** Adrenal incidentaloma, size, management, follow-up

## Abstract

The size of adrenal incidentalomas has important implications for diagnosis and management. Recommendations from endocrine societies do not all correlate with regard to adrenal incidentaloma size. Therefore, the aim was to compare adrenal incidentaloma size recommendations between different endocrine societies and the reasoning for these recommendations.

Eight different international guidelines were reviewed and compared. The smaller the size of the incidentaloma, the lower the risk for malignancy. The majority of guidelines consider 4 cm as the cut-off, but there are discrepancies. Size indications for laparoscopic adrenalectomy have a wide range from less than 4 cm up to 12 cm. The follow-up period of adrenal incidentalomas, as well as what is considered significant growth over that period, varies between the recommendations.

Therefore, the clinician should be aware of the differences when managing a patient with adrenal incidentaloma. There are discrepancies in size considerations with regard to significance, treatment options, optimal follow-up period, and further management.

**Main Points**

- All adrenal incidentalomas with clinically significant hormone secretion are recommended for adrenalectomy regardless of size.
- The majority of endocrine societies agree that adrenal incidentalomas less than 4 cm are considered low risk for malignancy.
- There are discrepancies regarding the size (varying from 4 to 12 cm) and appropriate operative modality for unilateral non-functional adrenal incidentalomas that are suspicious for malignancy.
- There is controversy regarding the size of adrenal incidentalomas and the appropriate follow-up time.
- There is controversy regarding the size of adrenal incidentalomas and functionality.

## Introduction

An adrenal incidentaloma is defined as an asymptomatic mass discovered on imaging investigations that were originally ordered for any reason other than adrenal disease.^1^ The definition excludes adrenal masses discovered during imaging investigations for tumor staging of extra-adrenal malignancies and for adrenal masses discovered during the screening that are associated with hereditary syndromes.^1^ Adrenal incidentalomas are diagnosed up to 7% in patients over the age of 70 years and even more rarely in patients less than 40 years.^2,3^ The 2 main concerns of an adrenal incidentaloma are morphological and functional status. Adrenal incidentalomas are commonly benign adenomas,^1^ with the prevalence of malignancy reported between 1.9% and 4.7%.^4^ Adrenal incidentalomas are commonly nonfunctioning adenomas (80%).^3,5^ Majority of adrenal hypersecretion are due to autonomous cortisol secretion, 1%-29%; followed by pheochromocytoma, 1.5%-14%; and aldosterone-secreting tumors, 1.6%-3.3%.^1,6^ Adrenal incidentalomas are not consistently investigated as recommended by international guidelines.^7-10^ A large component to diagnose and manage an adrenal incidentaloma is based on the size of the tumor. With regard to adrenal incidentaloma size, there is controversy regarding incidentaloma significance, treatment, optimal follow-up period, and further management. Therefore, the aim was to compare adrenal incidentaloma size recommendations between different endocrine societies and the reasoning for these recommendations.

## Clinical and Research Consequences

### Size of Adrenal Incidentalomas

Adrenal masses less than 1 cm are not considered a true adrenal incidentaloma and therefore not considered for further diagnostic work-up unless there are clinical features of excess adrenal hormone production.^4,11^ In 2002, the National Institute of Health (NIH) consensus considered adrenal tumors less than 4 cm as a low risk of malignancy, 4-6 cm as indeterminate, and greater than 6 cm as a high risk of malignancy.^12^ They stated that the prevalence of adrenocortical carcinoma was 2% in tumors up to 4 cm, 6% in tumors greater than 4 to 6 cm, and 25% in tumors larger than 6 cm.^12^ However, the NIH acknowledged the limitation of clinical data on the prevalence and natural history of incidentalomas.

More recent recommendations have been published including the 2016 European Society of Endocrinology (ESE) Clinical Practice Guideline in collaboration with the European Network for the Study of Adrenal Tumors (ENSAT), the 2009 American Association of Clinical Endocrinologists (AACE) and the 2009 American Association of Endocrine Surgeons (AAES) Medical Guidelines, the 2017 Korean Endocrine Society (KES), the 2011 Canadian Urological Association (CUA) and the 2011 Italian Association of Clinical Endocrinologists, or Associazione Medici Endocrinologi (AME).^1,5,11,13,14^ These endocrine societies agree, like the NIH, that nonfunctioning adrenal incidentalomas less than 4 cm are considered as low risk. However, in contrast to the NIH of 6 cm as high risk, the ESE, ENSAT, AACE, AAES, KES, CUA, and AME all agree that adrenal incidentalomas with a size of 4 cm or greater have a higher risk of malignancy and therefore qualifies for surgery.^1,5,11,13,14^ The panel, from the ESE and ENSAT, acknowledges the guideline of 4 cm is only based on expert opinion and not from documented clinical research.^1^ A national Italian study reported a diameter of 4 cm has a sensitivity of 93% to diagnose malignancy but with a low specificity at 24%.^5,15^ A difference was seen in a Korean study which documented a diameter of 4.75 cm as an accurate size to determine malignant adrenal tumors.^16^ In contrast, a radiological study reported the mean diameter of malignant adrenal lesions was 2.3 cm with a range of 1 to 4.1 cm.^17^. Although the majority of adrenocortical carcinomas are greater than 4 cm, the majority (60%) of non-adrenocortical malignant tumors, including lymphomas and metastases, are less than 4 cm in size with a median of 3 cm.^18,19^ Up to 15% of adrenal tumors that are less than 4 cm may be malignant.^20^

### Management

All adrenal incidentalomas with clinically significant hormone secretion are recommended for adrenalectomy.^1^ For nonfunctioning adrenal incidentalomas, all the endocrine societies agree that diameter less than 4 cm does not require intervention if they have benign signs on non-contrasted computed tomography (CT) evaluation, that is, Hounsfield units (HU) ≤10, smooth borders, and homogenous density.^1,6^ According to the ESE and ENSAT, adrenal incidentalomas less than 4 cm with suspicious features on non-contrasted CT evaluation, that is, HU ≥10, irregular borders, and heterogeneous density, should have either an additional imaging modality, follow-up in 6-12 months, or undergo adrenalectomy.^1,5^

There are discrepancies regarding the size and operative modality for unilateral adrenalectomy, that is, laparoscopic versus open. Different cut-off diameters have been documented for transperitoneal laparoscopic adrenalectomy. The National Comprehensive Cancer Network (NCCN) clinical practice guidelines recommend laparoscopic adrenalectomy up to 4 cm.^21^ The ESE and ENSAT recommend laparoscopic adrenalectomy be performed up to a cut-off diameter of 6 cm.^1^ The NCCN, ESE, and ENSAT do not advocate for laparoscopic adrenalectomy in the presence of a diagnosed adrenocorticoid carcinoma or when there are signs of local infiltration in the presence of suspicion of incidentaloma malignancy.^1,21,22^ However, other reports advocate even in the presence of adrenocortical carcinoma (excluding stage 4 malignancy) adrenal tumors up to 10 cm in diameter can be removed laparoscopically.^27^ Furthermore, studies have even demonstrated successful laparoscopic excision of adrenal tumors up to 12 cm.^23^

The larger the diameter of the tumor, the greater the technical challenges associated with adrenalectomy.^23^ The size of the adrenal lesion and the decision to perform laparoscopic adrenalectomy is also influenced by the site. In comparison to the transperitoneal approach, retroperitoneal laparoscopic adrenalectomy is appropriate up to a diameter of 6 cm.^23^ Retroperitoneal approach has disadvantages due to the inability to perform other abdominal procedures or explore the peritoneal cavity.^23^

Laparoscopic resection for malignant tumors has a higher risk of port site seeding, local recurrence, and peritoneal dissemination.^24,25^ Laparoscopic adrenalectomy also has a significant shorter time of recurrence, by almost 10 months, and a higher risk of positive resection margins or intraoperative tumor spillage compared to open surgery.^22,26^ Overall survival has also been shown to be longer for open surgery in those with malignant adrenal tumors, specifically stage 2.^26^ By contrast, other studies have reported no change or non-inferiority with laparoscopic compared to open adrenalectomy.^27,28^ Due to the conflicting reports, the experience of the surgeon to perform a laparoscopic adrenalectomy should also be taken into consideration.

### Follow-Up and Size

Progressive growth of the incidentaloma is concerning in follow-up investigations. Up to 37% of incidentalomas may demonstrate an increase in size on follow-up investigations.^29^ Conversely, up to 20% may even demonstrate a decrease in size by more than 5 mm on follow-up.^29^ There is controversy regarding the size and follow-up of patients with adrenal incidentalomas.

Benign adrenal tumor growth is typically slow and insignificant,^30^ 2 mm growth over 52.8 months,^30^ Rarely, benign tumors may demonstrate a larger increase in size over a shorter period, that is, 10-20 mm over 3 years.^31^ The ESE and ENSAT do not recommend further follow-up if the initial investigations demonstrated an incidentaloma less than 4 cm with benign findings and biochemically no hormone hypersecretion.^1^ A disadvantage of not following up on these patients is the possibility of missing tumors that may increase in size above the cut-off value over time. Morelli et al^32^ therefore advocate the continued follow-up of patients with adrenal incidentalomas even if less than the initial cut-off diameter.

The ESE and ENSAT do recommend that indeterminate adrenal incidentalomas less than 4 cm, who did not undergo adrenalectomy initially, have a follow-up imaging after 6-12 months with either a non-contrasted CT or magnetic resonance imaging to assess the growth.^1^ Adrenal malignancy or metastasis would most likely increase in size during this period.^1^ According to the AACE and AAES, all patients that do not have adrenalectomy initially should have follow-up imaging at 3-6 months then annually for 1 to 2 years.^5^ The rationale for the frequent follow-up is the cumulative risk of tumor enlargement of 6% at 1 year, 14% at 2 years, and 29% at 5 years.^10,33^ The AME also recommends follow-up imaging at 3-6 months.^11^ The KES recommends a follow-up period of 1 year for all benign non-functional incidentalomas less than 2 cm. Similar to the AACE and AAES, adrenal incidentalomas less than 4 cm with indeterminate non-contrasted CT signs, the KES recommends 3-6 months follow-up initially, then 1-2 years, and thereafter for 4-5 years.^13^

The growth of the adrenal incidentaloma, or increase in size, which is considered significant and warrants adrenalectomy is also controversial. The ESE and ENSAT advocate the use of the Response Evaluation Criteria in Solid Tumors (RECIST) version 1.1 criteria in deciding what size is considered as progressive disease on follow-up.^1^ The RECIST criteria considers significant growth as an increase in the largest diameter by more than 20%, in combination with a 5 mm increase in the same diameter.^34^ Although the RECIST 1.1 criteria are used in multiple oncological trials, it has not been validated for adrenal tumors, but, according to expert opinion (ESE and ENSAT), can be adapted for incidentalomas.^1^ The increase in diameter of more than 5 mm on follow-up evaluations has also been agreed by other authors,^10^ including the CUA,^14^ and has been reported to occur in 17.4% at 2 years.^35^ The AACE, AAES, AME, and KES recommend an increase in diameter of more than 1 cm, which occurs in 20%, should be considered as significant growth.^5,13,11,33^ Another discrepancy of growth in diameter of more than 8 mm has been reported to be the predictor of malignancy, with a sensitivity of 72% and a specificity of 81.1%.^36,37^

When considering the size and functional status, the AACE and AAES recommend the hormonal work-up be repeated annually for 5 years in all patients, regardless of size.^5^ The rationale for the frequent follow-up is the cumulative risk of hormonal changes of 17% at 1 year, 14% at 2 years, and 47% at 5 years.^33^ Also, an incidentaloma with a diameter of 3 cm or greater has a higher risk of hormone hypersecretion.^33^ Hormone hypersecretion is usually asymptomatic and may only peak 3 to 4 years after initial detection of the incidentaloma.^33^ However, the ESE and ENSAT do not recommend repeating the hormonal investigations when the initial work-up did not demonstrate a hyperfunctioning tumor.^1^ The explanation is the low risk of developing Cushing’s syndrome at 0.1% regardless if the original hormonal investigations showed an autonomous cortisol secretion or a nonfunctioning adrenal incidentaloma.^30^ There are exceptions though such as patients with worsening of comorbidities, such as hypertension, or clinically have new features of adrenal hypersecretion, then repeating the hormonal work-up is reasonable.^1^ Also, patients with no signs of Cushing’s syndrome, but have worsening co-morbidities due to raised cortisol levels, can have an annual cortisol level assessment.^1^

## Conclusion

The clinician should be aware of the differences between the various international endocrine societies when managing a patient with adrenal incidentaloma. There are discrepancies in size considerations with regard to significance, treatment options, optimal follow-up period, and further management. The clinician should be aware of the reasoning for the various size recommendations.
